# Evaluation of Implementation Effect of Cervical Cancer Comprehensive Treatment Patients With Whole-Course High-Quality Care Combined With Network Continuation Care

**DOI:** 10.3389/fsurg.2022.838848

**Published:** 2022-01-31

**Authors:** Jing Chen, Hui Bai

**Affiliations:** ^1^Department of Gynaecology, The First Affiliated Hospital of Jinzhou Medical University, Jinzhou, China; ^2^Department of Nephrology, The First Affiliated Hospital of Jinzhou Medical University, Jinzhou, China

**Keywords:** cervical cancer, comprehensive treatment, whole-course high-quality care, network continuation care, implementation effect

## Abstract

**Purpose:**

Discuss the implementation effect of cervical cancer comprehensive treatment patients applying whole-course high-quality care combined with network continuation care.

**Methods:**

From August 2020 to August 2021, 120 patients who met the inclusion criteria for comprehensive treatment of cervical cancer were divided into the regular group (*n* = 60) who received conventional care and the joint group (*n* = 60) who received whole-course high-quality care combined with network continuation care, according to the method of care. The comprehensive treatment cognition level, comprehensive treatment compliance, adverse reaction rate, quality of life questionnaire (QLQ-C30) score, self-rating anxiety/depression scale (SAS/SDS) score, and nursing satisfaction were compared between the two groups.

**Results:**

After care, the comprehensive treatment cognition score and comprehensive treatment compliance score were higher in the joint group than in the regular group (*P* < 0.05). After care, the incidence of radiation cystitis and radiation proctitis was lower in the joint group than that in the regular group (*P* < 0.05). After care, QLQ-C30 scores on symptom domains, functional domains, and single questions were higher in both groups than before care, and were higher in the joint group than in the regular group (*P* < 0.05). After care, SAS and SDS scores were lower in both groups than before care, and were lower in the joint group than in the regular group (*P* < 0.05). After care, the joint group was more satisfied with care than the regular group (*P* < 0.05).

**Conclusion:**

The implementation of cervical cancer comprehensive treatment patients with whole-course high-quality care combined with network continuation care has an ideal implementation effect, which can significantly increase the patient's cognition and compliance with treatment, the incidence of adverse reactions is less, the quality of life and emotional state have also improved significantly, and care satisfaction has also increased accordingly.

## Introduction

Cervical cancer is a malignant tumor of the cervical canal and uterus and vagina associated with multiple factors such as human papillomavirus (HPV) infection, unclean sex, smoking, young age at first birth, prolificacy, immunosuppression, etc. (1, 2). Squamous carcinoma, adenocarcinoma, and adenosquamous carcinoma are their common pathological types (3). Its *in situ* carcinoma is more likely to occur in women aged 30–35 years, and invasive carcinoma is more likely to occur in women aged 45–55 years, and both of which have a tendency to develop at a lower age in recent years (4). The earlier the disease is treated and the earlier the clinical stage, the higher the survival rate of patients. The current 5 year survival rate worldwide is about 55.50%, which can pose a great threat to the life safety and physical and mental health of patients (5). A comprehensive treatment plan based on surgery and radiotherapy, supplemented by chemotherapy, is usually adopted clinically after considering the patient's age, physical condition, fertility needs, and cancer stage. However, there is a high risk of complications such as pain, radiation cystitis, radiation proctitis, bone marrow suppression, skin damage, hair loss, and gastrointestinal symptoms after surgery or during radiotherapy or chemotherapy, which affects patients' physical and mental status and treatment compliance (6, 7). However, the lack of scientific medical guidance and standardized nursing management after patients leave the hospital makes them more prone to a series of near and long-term complications and sequelae. Therefore, how to improve the physical and mental status and treatment compliance of cervical cancer patients, enhance the quality of post-treatment survival, and reduce the occurrence of adverse reactions has become an urgent issue for medical professionals to address. The whole-course high-quality care is to give maximum high-quality nursing support in the whole stage of patients' admission, protect patients' personal privacy and respect patients' dignity and value, so as to maximize the treatment effect and minimize adverse reactions. The network continuous care is an out of hospital extension of in-hospital nursing service with wechat and other information tools as communication media to help patients solve nursing problems after discharge. It has the characteristics of low cost and high efficiency. In recent years, our department has applied the whole-course high-quality care combined with network continuation care to the care service of cervical cancer comprehensive treatment patients, and the effect is ideal, which is reported as follows.

## Materials and Methods

### Research Object

From August 2020 to August 2021, 120 patients with cervical cancer who met the inclusion criteria were enrolled in this study. Inclusion criteria: All met the diagnostic criteria of American Society for Colposcopy and Cervical Pathology for cervical cancer (8) and the staging criteria of International Federation of gynecology and obstetrics for cervical cancer (9); Clinical stage I–III; All received comprehensive anti-cancer treatment in our hospital; Junior high school education and above; Proficiency in the application of WeChat; All met the follow-up conditions. Exclusion criteria: Previous neurological or psychiatric history; Pregnancy and lactation; Presence of significant organ damage; Presence of malignancy at other sites; Presence of cognitive and communication impairment. All study subjects were divided into the regular group (*n* = 60) and the joint group (*n* = 60) according to the different methods of care. There was no significant difference in age, clinical stage, type of pathology, and education level between the two groups, which were comparable (*P* > 0.05) (Table 1).

**Table 1:** Comparison of baseline information between the two groups (*n*, $\bar{x}$±s, %).

**Items**	**Regular group (*n* = 60)**	**Joint group (*n* = 60)**	***t*/χ^2^ value**	***P-*value**
Age (years old)	49.42 ± 6.49	50.24 ± 6.49	0.703	0.483
Clinical stage (*n*, %)			0.586	0.746
Period I	18 (30.00)	20 (33.33)		
Period II	23 (38.33)	19 (31.67)		
Period III	19 (31.67)	21 (35.00)		
Type of pathology (*n*, %)			1.097	0.578
Squamous carcinoma	45 (75.00)	48 (80.00)		
Adenocarcinoma	14 (23.33)	10 (16.67)		
Adenosquamous carcinoma	1 (1.67)	2 (3.33)		
Education level (*n*, %)			0.391	0.532
Middle School–High School	46 (76.67)	43 (71.67)		
University and above	14 (23.33)	17 (28.33)		

### Research Methods

Both groups received comprehensive treatment for cervical cancer. That was, for patients with stage Ia1–Ib1 cervical cancer, the cervix or uterine body should be surgically removed as appropriate + pelvic lymph node dissection and/or paraaortic lymph node dissection, for those with high-risk factors, adjuvant radiotherapy and chemotherapy; For patients with stage Ib2–IIa2 cervical cancer, radical hysterectomy + pelvic lymph node dissection and/or paraaortic lymph node dissection and radiotherapy and chemotherapy were performed; For patients with stage IIb–IIIb cervical cancer, concurrent chemoradiotherapy with radiotherapy as the core was implemented. On this basis, the regular group received conventional care interventions and the joint group received whole-course high-quality care combined with network continuation care interventions.

Conventional care: In other words, according to the comprehensive treatment process of cervical cancer, patients were given verbal health education about cervical cancer; they were informed of the precautions and examination items during surgery and radiotherapy; they were advised to complete daily basic care; they were strictly supervised to take medication and receive treatment on time; the hygiene and room temperature of wards and treatment rooms were maintained; and the changes of patients' vital signs were strictly monitored and recorded.

Whole-course high-quality care: ①Before comprehensive treatment: Medical and nursing staff should actively communicate with patients and their families, patiently inform patients or their families of the rationale, necessity, effects and possible adverse reactions of the treatment. Through conversations, letting them fully understand the basic knowledge and precautions related to treatment, so that they had good self-management skills and ability to respond to emergencies. For example, radiotherapy maybe cause burning, redness, swelling and other damage to the skin of the patient's healthy parts. Therefore, medical staff should inform patients or their families in advance to prepare soft, cotton underwear to reduce contact with the skin of the affected area. For patients with psychological stress or their family members, they should be informed that anxiety, tension, fear, etc. are all normal psychological emotions, and should be addressed squarely and actively relieved. Through verbal communication, understand the reasons for the patients' negative emotions and provide targeted psychological counseling to make the patients face treatment with an optimistic attitude. ②During comprehensive treatment: Recording the patient's vital signs changes and adverse reactions in detail, and giving targeted treatment measures to them. In addition, strengthenning the screening of patients' pain and other problems, timely intervene in case of abnormality. At the same time, medical staff stilled need to pay close attention to the patient's psychological state and source of negative emotions. The discomfort of the patient or the psychological defense mechanism could be eliminated in time through methods such as psychological suggestion and attention shift, so as to obtain the cooperation and trust of the patient as much as possible, thereby improving the compliance to receive treatment. ③After comprehensive treatment: Actively dealing with treatment-related adverse reactions and complications. For example, for patients with skin burns, they should be told to keep their skin dry, keep away from light, do not touch vigorously, and do not use drugs without authorization. Patients with gastrointestinal symptoms should be encouraged to eat consistently, and medical staff can work with the patient's family to establish a scientific, reasonable and rich and diverse healthy diet for the patient. Instruct patients to increase their drinking water appropriately after radiotherapy and chemotherapy to accelerate the metabolism and excretion of drugs. Eat more fresh ingredients that can boost immunity every day, and try to maintain or enhance the flavor of the food during preparation to stimulate the appetite of the patient. It was also possible to reduce gastrointestinal discomfort in patients by eating small meals and frequent meals. For patients with radiation cystitis, gauze could be filled in the vagina to reduce radiation damage, and dexamethasone, gentamicin and other drugs could also be appropriately treated. For patients with radiation proctitis, bismuth carbonate should be taken orally in time, and the patients should be instructed to reduce the intake of crude fiber to reduce the secondary irritation to the intestinal tract. After the patient was discharged from the hospital, the researchers followed up by telephone twice a month to understand their physical and mental status and health problems, supervised patient's treatment progress and health management, etc., actively corrected patients' misunderstandings about disease and health, and patiently answered patients' health problems and treatment confusion.

Network continuation care: Using WeChat as the medium of implementation. First, the department established a WeChat group of network continuation care, and drew the members of the department into the group. Then posted the admission QR code in the department, and patients could scan the code to join the group upon admission. Arranging a full-time nurse to develop a treatment cycle schedule for each patient, so that patients can obtain information such as consultation appointments, discharge instructions, return treatment time, follow-up time, treatment progress, departmental contact information, and other relevant information within the group. At the same time, the group regularly pushed related articles about common complications and treatment methods after cervical cancer radiotherapy and chemotherapy, and dietary precautions, which patients could download and read by themselves. Every day, a physician and a full-time nurse were arranged to provide professional answers and guidance to patients' doubts and problems. Questions related to personal privacy such as sex life could be reported and answered privately. At the same time, patients could also communicate or give feedback on their own in the group, share their anti-cancer experience or nursing improvement opinions, etc. The department held a forum once a month to summarize the problems and feedback collected and discuss solutions, so as to continuously improve the group management process and service process of network continuation care.

### Observation Index

(1) Comprehensive treatment cognitive score: After care, the “Questionnaire for Comprehensive Treatment Cognitive Level of Cervical Cancer,” which was developed by our hospital, was used for assessment. The total score was 0–100, and the higher the score, the higher the cognitive level.

(2) Comprehensive treatment compliance score: After care, the “Questionnaire for Comprehensive Treatment Adherence of Cervical Cancer,” which was developed by our hospital, was used for assessment. The total score was 0–100, the higher the score, the better the compliance.

(3) Incidence of adverse reactions: The treatment-related adverse reactions such as radiation cystitis, radiation proctitis, bone marrow suppression and digestive symptoms during care were recorded in both groups.

(4) Quality of life questionnaire (QLQ-C30) score: Before and after care, the QLQ-C30 was used to assess the overall quality of life of both groups, including symptom domains, functional domains, and single-item problems. The total score of each item was 0~100, and the higher the score, the better the quality of life.

(5) Self-rating anxiety/depression scale (SAS/SDS) score: Before and after care, the emotional status of both groups was assessed by SAS/SDS. The former group was classified as having anxiety symptoms with a standard score of ≥50, and the latter group was classified as having depressive symptoms with a standard score of ≥53.

(6) Nursing satisfaction: After nursing care, the “Nursing Satisfaction Questionnaire for Cervical Cancer Patients,” which was developed by our hospital, was used for assessment. The total score of 90–100 was very satisfied, 60–89 was satisfied and <60 was unsatisfied.

### Statistical Methods

SPSS 22.0 software was applied, and the measurement data were expressed as mean ± standard deviation and compared by *t*-test. Count data were expressed as ratio, and the χ^2^ test was used for comparison. *P* < 0.05 was considered statistically significant.

## Results

### Comparison of Cognitive and Adherence Scores of Comprehensive Treatment of the Two Groups

**Figure 1:**
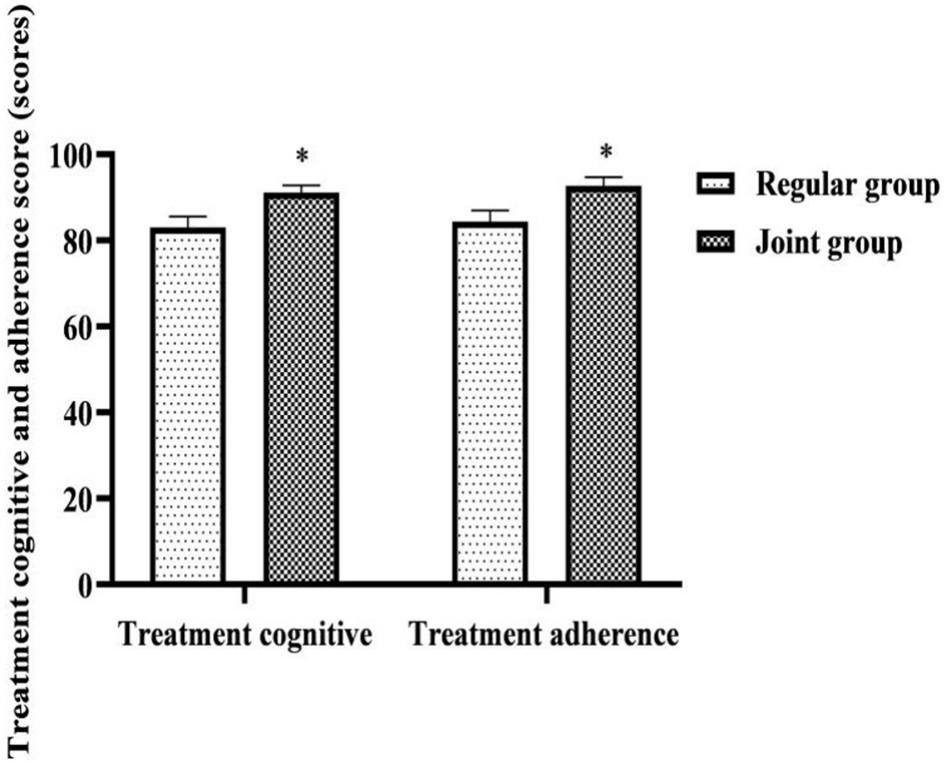
Comparison of cognitive and adherence scores of comprehensive treatment of the two groups. Compared with the same item in the regular group, *P < 0.05.

After care, the comprehensive treatment cognition score and comprehensive treatment compliance score were higher in the joint group than in the regular group (*P* < 0.05) (Figure 1).

### Comparison of the Incidence of Adverse Reactions of the Two Groups

After care, the incidence of radiation cystitis and radiation proctitis was lower in the joint group than that in the regular group (*P* < 0.05) (Table 2).

**Table 2:** Comparison of the incidence of adverse reactions of the two groups (*n*, %).

**Groups**	**Radiation cystitis**	**Radiation proctitis**	**Digestive symptoms**	**Bone marrow suppression**	**Others**
Regular group (*n* = 60)	14 (23.33)	10 (16.67)	22 (36.67)	47 (78.33)	27 (45.00)
Joint group (*n* = 60)	3 (5.00)	2 (3.33)	18 (30.00)	40 (66.67)	18 (30.00)
*χ^2^*value	8.292	5.926	0.600	2.048	2.880
*P-*value	0.004	0.015	0.439	0.152	0.090

### Comparison of QLQ-C30 Scores of the Two Groups

**Figure 2:**
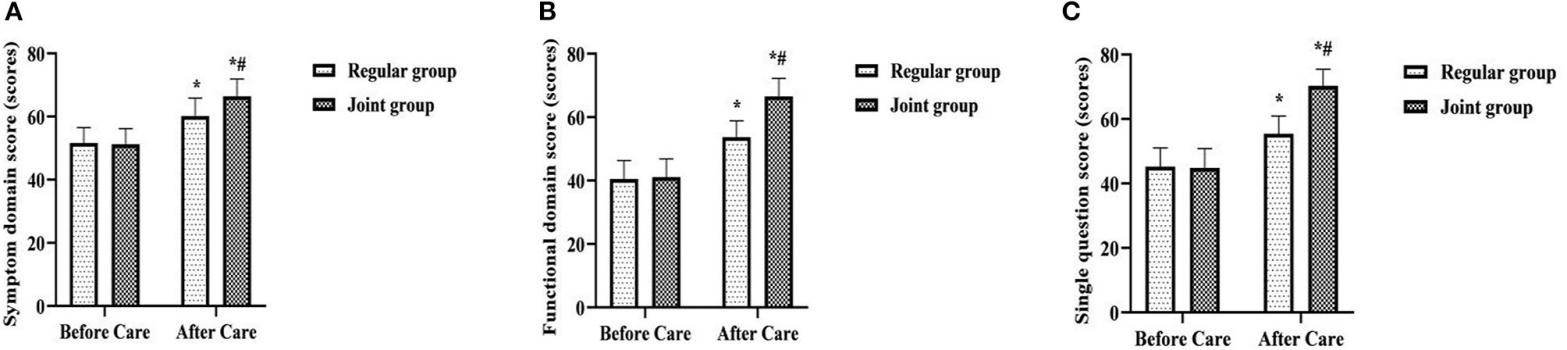
Comparison of QLQ-C30 scores of the two groups. **(A)** Symptom domain score; **(B)** Functional domain score; **(C)** Single question score. Compared with the same group before care, *P < 0.05; Compared with the regular group after care, ^#^*P* < 0.05.

After care, QLQ-C30 scores on symptom domains, functional domains, and single questions were higher in both groups than before care, and were higher in the joint group than in the regular group (*P* < 0.05) (Figure 2).

### Comparison of SAS and SDS Scores of the Two Groups

**Figure 3:**
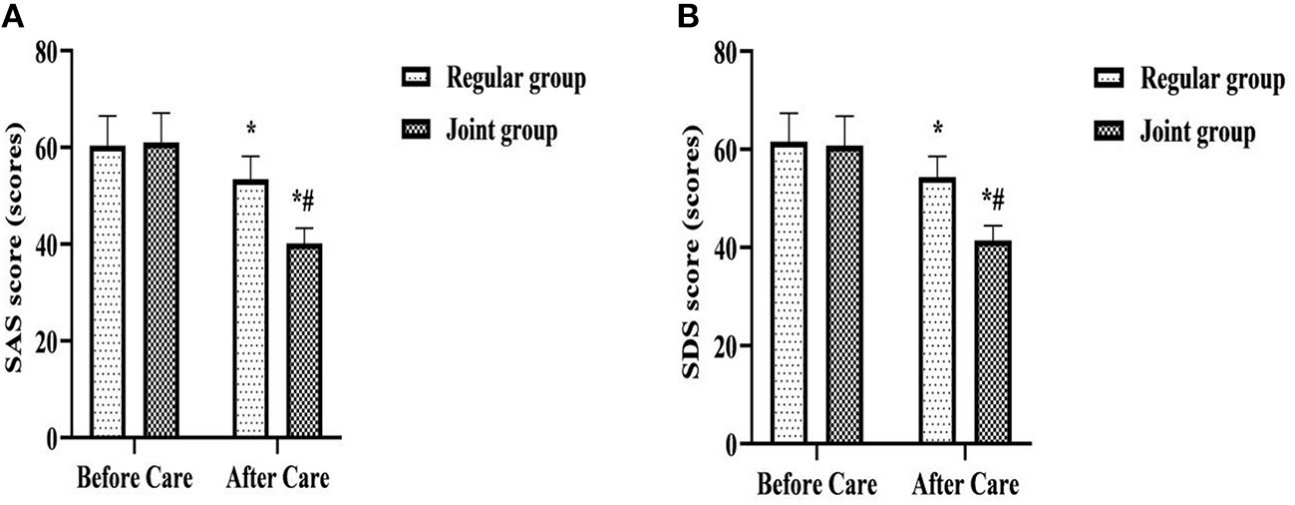
Comparison of SAS and SDS scores of the two groups. **(A)** SAS score; **(B)** SDS score. Compared with the same group before care, *P < 0.05; Compared with the regular group after care, ^#^*P* < 0.05.

After care, SAS and SDS scores were lower in both groups than before care, and were lower in the joint group than in the regular group (*P* < 0.05) (Figure 3).

### Comparison of Nursing Satisfaction of the Two Groups

After care, the joint group (96.67%) was more satisfied with care than the regular group (83.33%) (*P* < 0.05) (Table 3), 60–89 was satisfied and <60 was unsatisfied.

**Table 3:** Comparison of nursing satisfaction of the two groups (*n*, %).

**Groups**	**Very satisfied**	**Satisfied**	**Unsatisfied**	**Overall satisfaction rate**
Regular group (*n* = 60)	22 (36.67)	28 (46.67)	10 (16.67)	50 (83.33)
Joint group (*n* = 60)	37 (61.67)	21 (35.00)	2 (3.33)	58 (96.67)
*χ^2^*value				5.926
*P-*value				0.015

## Discussion

In recent years, with the popular use of HPV vaccine and cervical cytology screening, the incidence of cervical cancer and its precancerous lesions has been greatly reduced by early prevention, diagnosis and treatment, but patient survival is still low (10). In the implementation of comprehensive treatment for cervical cancer patients, the clinical approach mostly adopts staged treatment. That is, cervical cancer patients with stage Ia1–IIa2 are treated with comprehensive treatment measures mainly with surgery and supplemented with radiotherapy; patients with stage ≥IIb are treated with simultaneous radiotherapy and chemotherapy with radiotherapy as the core (7, 11). However, it has been observed that after cervical cancer surgery, the sense of experience such as pain or incompleteness caused by surgical resection can lead to a range of negative emotions such as low self-esteem, loss, and depression (12, 13); Damage to adjacent organs, fatigue or bone marrow suppression caused by radiotherapy can cause further damage to the patient's health status and quality of life (14, 15); Moreover, the damage to the digestive tract caused by chemotherapy can lead to decreased appetite, nausea and vomiting, and even rejection of food, and the patient's nutrient intake is insufficient, and the body's immunity can be further reduced (16, 17). These problems can lead to non-compliance or intolerance of patients to later radiotherapy and chemotherapy, and patients end up in the unfavorable situation of short survival time and low survival probability because they cannot complete their treatment. In addition, according to our clinical observation, under the conventional nursing mode, medical staff lack advance judgment and prevention of patients' treatment adverse reactions or negative emotions, so they often fall into a panic and passive situation when problems appear, which is not conducive to the maintenance of nurse-patient relationship and the establishment of nurse-patient trust. Based on the above, it is obviously not enough to provide only routine nursing support for patients receiving comprehensive treatment for cervical cancer, and more humane and comprehensive high-quality nursing services are urgently needed to be implemented.

This study implemented the whole-course high-quality care combined with network continuation care for patients with comprehensive treatment of cervical cancer. Both of them follow the concept of “patient-centered” and strive to provide quality and humanized nursing care throughout the whole stage of in-hospital and out-of-hospital treatment. The implementation results show that after care, the comprehensive treatment cognition score and comprehensive treatment compliance score were higher in the joint group than in the regular group; the incidence of radiation cystitis and radiation proctitis was lower in the joint group than that in the regular group; QLQ-C30 scores on symptom domains, functional domains, and single questions were higher in both groups than before care, and were higher in the joint group than in the regular group (*P* < 0.05). In clinical care, almost all patients have varying degrees of lack of basic knowledge related to treatment (18). This may lead to poor treatment cooperation or improper self-care, which ultimately aggravates the occurrence of treatment-related complications or adverse effects and affects the quality of life and health status of patients after treatment (19). During the whole-course high-quality care in this study, before comprehensive treatment, medical staff explained to the patient the principle, necessity, effect and possible adverse reactions of the treatment. This was not only an early prediction and prevention by medical staff of possible problems during and after comprehensive treatment of patients, but also helped patients to be psychologically prepared. In order to reduce the occurrence of unfavorable expectations, patients entered the preparation state in advance to cooperate with medical staff for treatment and nursing, and actively learned basic knowledge and precautions related to treatment. Furthermore, its treatment cognition level, treatment compliance, self-management ability and emergency response ability had been greatly improved, which would ultimately help reduce treatment-related adverse reactions and improve the prognostic quality of life. During the comprehensive treatment, medical staff not only paid attention to the targeted care of the patient's existing vital signs changes and various adverse reactions, but also paid attention to the comprehensive screening and protection of the patients' possible problems. Early detection and treatment of the direct or indirect damage that surgery or radiotherapy and chemotherapy might bring to the body would also help patients maintain their quality of life and health. After comprehensive treatment, the focus of medical staff's care was to actively treat complications and maintain the treatment effect. After the comprehensive treatment in this study, the patient's daily diet and daily life were intervened by combining with the patient's family members. The family members could play an auxiliary role in helping, supervising and reminding. This could better meet the treatment needs of patients and enhance their confidence in disease resistance, which would ultimately help improve the anti-cancer effect of comprehensive treatment. In addition, the application of network continuation care in patients' home treatment, which provided information, consultation and out-of-hospital reminders to patients with the interactive information function of WeChat. This made patients no longer limited to the time and space constraints of inconvenient to answer the phone, unclear speech, or unable to understand the content of the communication in a timely manner under the conventional care model, it had also changed the past model that patients need to integrate and screen effective information to cooperate with treatment during home treatment on their own. Medical staff could also learn about the patient's disease dynamics and treatment needs in real time through the patient's condition feedback and consultation questions, and updated and improved the nursing content accordingly. This helped to maintain the coordination and continuity of care during home treatment of patients, and was an effective way to foster patients' healthy self-reflection behavior and ensure patient treatment compliance and safety.

When the patient has misunderstandings about the disease or treatment, it can not only affect the treatment compliance and treatment effect, but also easily cause psychological imbalance, leading to a series of bad emotions such as anxiety, depression, worry, resistance, etc., which in turn can also affect the patient's compliance and effect with treatment (20). After care in this study, SAS and SDS scores were lower in both groups than before care, and were lower in the joint group than in the regular group; After care, the joint group was more satisfied with care than the regular group (*P* < 0.05). It is suggested that whole-course high-quality care combined with network continuation care is an effective initiative to improve patients' psychological stress level and nursing satisfaction, and to maintain patients' good therapeutic mindset. Analyzing the reasons, all stages of the whole-course high-quality care in this study focused on the control and enlightenment of patients' psychological state. Among them, the meticulous care and patient guidance of medical staff, and the involvement of family relationships are all helpful to the establishment of patients' confidence in treatment and the relief of negative emotions. In addition, in network continuation care, patients can discuss embarrassing problems encountered in daily life without having to meet with medical staff, which is an effective way to relieve patients' psychological burden and psychological defense mechanism, enhance nurse-patient trust and promote mutual understanding. It enables patients to communicate more candidly about psychological problems and receive psychological counseling from medical staff.

## Conclusion

To sum up, after the implementation of the whole-course high-quality care combined with network continuation care, patients with comprehensive cervical cancer treatment have greatly improved their cognition and compliance with treatment, and treatment-related side effects have been greatly reduced. This is helpful to the overall solution of the patients' physical, psychological and social problems, so their quality of life is significantly improved, and the patient's nursing satisfaction is high.

## Data Availability Statement

The original contributions presented in the study are included in the article/supplementary material, further inquiries can be directed to the corresponding author/s.

## Ethics Statement

The studies involving human participants were reviewed and approved by the First Affiliated Hospital of Jinzhou Medical University. The patients/participants provided their written informed consent to participate in this study.

## Author Contributions

JC is mainly responsible for research results testing, data statistics, and paper writing. HB is mainly responsible for research design and guidance of the entire research process. Both authors contributed to the article and approved the submitted version.

## Conflict of Interest

The authors declare that the research was conducted in the absence of any commercial or financial relationships that could be construed as a potential conflict of interest.

## Publisher's Note

All claims expressed in this article are solely those of the authors and do not necessarily represent those of their affiliated organizations, or those of the publisher, the editors and the reviewers. Any product that may be evaluated in this article, or claim that may be made by its manufacturer, is not guaranteed or endorsed by the publisher.
